# The Influence of Support Groups on Quality of Life in Head and Neck Cancer Patients

**DOI:** 10.5402/2011/250142

**Published:** 2011-11-03

**Authors:** Sarah E. Mowry, Marilene B. Wang

**Affiliations:** Division of Head and Neck Surgery (Otolaryngology), David Geffen School of Medicine at the University of California, Los Angeles, CA 90095-1624, USA

## Abstract

*Objective*. To compare quality of life in head and neck cancer (HNC) patients following treatment. *Methods*. The Short Form-36 Version 2 (SF-36v2) was utilized to measure patient quality of life. *Results*. For all 8 parameters measured by the SF-36V2, HNC patients had lower mean scores than the US population means. Support group patients had significantly worse scores than US population norms in role-physical, social functioning, and role-emotional. There were no significant differences between support group and control patients for the 8 parameters measured by the SF-36v2. *Conclusions*. HNC patients report significantly worse quality of life than US population norms in several physical and emotional areas. Our study did not demonstrate improved quality of life for support group patients. The increased incidence of oropharyngeal cancer and chemotherapy treatment in the support group patients in our study were factors which were likely to have lowered the overall scores in these patients.

## 1. Introduction

Approximately 43,000 new cases of head and neck cancer (HNC) are diagnosed each year in the United States. Despite changes in treatment regimens (i.e., organ preservation protocols and the addition of chemotherapy to primary radiation treatment), survival curves have not changed over the past 20 years. In this setting, there has been an increasing interest in trying to understand and maximize patient quality of life (QOL). 

Quality of life is a subjective perception by the patient that includes physical, emotional, and social well-being. It is well-established that different subsites within the head and neck have different perceived QOL following treatment; patients with oropharyngeal carcinoma tend to report worse quality of life than do other head and neck cancer patients and have poorer functional outcomes [1, 2]. Furthermore, specific aspects of treatment greatly affect a patient's quality of life. Terrell et al. identified a number of factors that have a negative impact on QOL including need for gastrostomy and tracheostomy tubes, comorbid medical conditions, addition of chemotherapy to treatment, and need for a neck dissection [3]. 

A variety of mechanisms have been proposed in the literature to improve patient quality of life, including educational programs, support group meetings, biofeedback therapy, and cognitive behavioral therapy. However, the impact of these interventions has not been extensively studied in head and neck cancer patients. 

Support group therapy is a well-established means of psychosocial intervention in the cancer literature. The majority of research regarding support groups comes from the breast cancer literature. However, only a handful of studies regarding support group therapy in the head and neck population exist, and the results are mixed [4, 5]. The current study was designed to compare HNC patients who participate in a support group to those who do not participate in a support group. These two groups were then combined and compared to normative US population data. We hypothesized that HNC support group patients would do better than their peers who do not participate and that both groups would have lower QOL scores than the US population.

## 2. Methods and Patients

The Institutional Review Board for Human Subjects at UCLA approved this study (IRB # G06-11-050-01). Patients were recruited in two ways: (1) directly from Support for People with Oral and Head and Neck Cancer (SPOHNC) support group meetings or (2) during cancer surveillance follow-up appointments in the clinic of a tertiary referral center. All patients were provided informed consent and a completed questionnaire implied their consent to participate in the study. 

Support for People with Oral and Head and Neck Cancer (SPOHNC) is a nonprofit organization run by HNC survivors to help other patients learn more about and cope with their disease. Local chapters sponsor support group meeting across the country. The chapter of SPOHNC affiliated with our institution is organized by a clinical social worker who also facilitates the support group discussions. 

Patients were asked to fill out the Medical Outcomes Study Short Form-36 Item Health Survey version 2 (SF-36v2, Quality Metric, Inc., Lincoln, RI) which asks questions regarding 8 domains related to global QOL. The domains investigated by this instrument are physical functioning (PF), role-physical (RF), bodily pain (BP), general health (GH), vitality (VT), social functioning (SF), role-emotional (RE), and mental health (MH). The instrument consists of 36 questions arranged in 8 sections. A higher subscale score represents a better perceived quality of life. Scores can range from 0 (very poor quality of life) to 100 (excellent quality of life). The PF domain measures ability to do specific activities, such as walking 100 yards or bathing oneself. The RP domain queries how any physical limitations have impacted the patient's life, such as cutting back on work hours or accomplishing less than expected. Bodily pain (BP) asks about the amount of pain and if it has limited activity. The general health (GH) domain measures the patient's perception of their health; questions include a rating of their health from excellent to poor. Other questions in this domain ask the patient to compare their health to others and their expectations for their health. Vitality (V) queries the patient's perception of their energy level, such as full of energy to “worn out.” Social functioning (SF) measures how much health-related issues have interfered with social activity. The role emotional (RE) domain asks the patient to assess how much these health-related issues have impacted the performance of activities. Mental health (MH) asks questions regarding anxiety and depression. The final question of the instrument asks the respondent to compare their current status to their health one year ago. 

Scoring of the SF-36v2 questionnaires was done according to the method previously described [6]. A supplemental questionnaire, designed by the authors, was also included, which asked patients to self-report the type and location of cancer, method of treatment, and time since completion of treatment. Patients were allowed to write any comments they wanted on this supplemental form. Data obtained from the questionnaires was then entered into a Microsoft Excel spreadsheet which was programmed to perform conversion calculations as described by the scoring instructions. SDSS software was then used to perform statistical analysis of the data. Two sets of data were generated: one comparing cancer patients to 1998 US norms and another comparing the different cancer groups to each other. When comparing cancer groups, both unadjusted and adjusted measurements were calculated. Adjustments were made for elapsed time since the end of treatment (in years), cancer therapies (including surgery, radiotherapy, and chemotherapy), and cancer location (oral cavity/oropharynx/other and larynx). A 1-sample *t*-test was performed to determine if the mean score differed significantly from the US norms. In the case of sparse data, the Fisher exact test was used to compare categorical variables with 2 levels and Monte Carlo simulation to compare categorical variables with more than 2 levels. Missing values were imputed using the average score based on questions that were answered on the corresponding SF-36v2 scale, but only if at least 50% of the questions were answered. Otherwise, the scale score was considered missing.

## 3. Results

A total of 37 patients completed the SF-36v2, 21 from the support group and 16 from the nonsupport group. Within the support group category, 12 were female (60%) and the average age was 61 years old. There was a statistically significant difference between the two groups as to the location of their primary tumor. Forty-five percent of patients in the support group reported an oropharyngeal primary site, while 68% of nonsupport group patients reported an oral cavity primary site (*P* = 0.0036). Both groups had undergone multimodality therapy; there was no statistically significant difference between the groups as to the type of therapy rendered (most patients had undergone surgery and/or radiation therapy with or without chemotherapy) (Table 1). Because the patients provided the information regarding their treatment regimens, specific data regarding radiotherapy dosing and exact chemotherapeutic regimens was not available.

### 3.1. All HNC Patients versus US Normative Values

All values for HNC patients were lower than the 1998 US normative values. However, only 4 domains were statistically significantly different: physical function, role physical, social function, and role emotional (*P* = 0.0087, 0.003, 0.0015, and 0.0005, resp.). For role physical, social function, and role emotional, greater than 20% of respondents reported scores 2 standard deviations below the 1998 normative means (Table 2). Furthermore, when analyzed separately, support group patients did significantly worse than US norms for physical function, role physical, social function, general health, and role emotional (Table 2).

### 3.2. Support Group Patients versus Cancer Control Patients

There were 21 patients in the support group cohort and 16 patients in the control cohort. There was no statistically significant difference for any domain between the two groups nor did any domain approach statistical significance (Table 3). As noted above, there were significant differences between the two groups with regard to primary cancer subsite.

## 4. Discussion

The diagnosis of head and neck cancer has a profound effect on patients. They are affected physically, emotionally, and socially; all of which lead to a change in their perceived global quality of life. In this study, we failed to find a statistically significant improvement in patients' perceived QOL when they participate in a head and neck cancer-specific support group. However, many support group participants anecdotally related the comfort they received by being able to speak to others who have very similar concerns and problems. While this aspect of the support group did not appear to impact their overall QOL, it is obviously important to many participants to feel they were not alone. Not surprisingly, cancer survivors reported decreased quality of life for all domains; however, only about half of the queried domains were statistically significantly lower. 

The support group at our institution is sponsored by SPOHNC. The local chapter of this national group provides a monthly forum for discussion. These meetings are held at the hospital, and medical professionals are frequently invited to come to the discussion to talk about recent advances in head and neck cancer or treatment-related issues. This group has been active for a number of years at our institution and participants in the study were recruited directly from the meeting by the authors. A number of the group participants have been regularly active members for many years, while others were relatively new to the support group forum. Therefore, the range of time from treatment to questionnaire was highly variable in the support group cohort of this study. However, we feel that recruiting patients directly from a previously constituted support group may more accurately reflect the effect of the support group on those who participate. Other studies in the literature have looked at the QOL of patients who were asked to participate in a support group. Two previous studies created support groups for the patients to participate in, rather than drawing data from patients in preexisting support groups [4, 5]. 

The literature regarding the effect of support group therapy on head and neck patients is somewhat sparse, but there is a fairly large body of work regarding support group participation in patients with breast, lung, and colon cancer. Support groups have been found to decrease anxiety and depression [7]. While the results in other cancer groups demonstrate substantial improvement in patients' perceived QOL, our study failed to find a significant difference in QOL outcomes between head and neck support group participants and nonparticipants. This finding was unexpected. Several explanations may exist for this lack of difference. First, perhaps the difference in cancer subsite affected the outcome. Support group participants were more likely to have oropharyngeal carcinoma, while nonparticipants were more likely to have oral tongue lesions. It is generally agreed that patients with oropharyngeal carcinoma have somewhat poorer QOL outcomes than other subsites. Thus, this bias toward poorer outcomes may have negated the positive effect of the support group on their QOL. The second explanation may lie with the fact that the study group was drawn from a preexisting support group. We did not test for underlying depressive disorders or question patients regarding their psychological coping mechanisms. Perhaps those patients who participate regularly in a support group lack some of these coping mechanisms and therefore need the psychological support of the group more than their nonparticipating counterparts. Further research into the area is necessary to determine if support group therapy would benefit specific cancer subsite patients differently. It is also possible that different subsites of head and neck cancer require different methods or styles of supportive therapy. Lastly, perhaps the choice of instrument affected the ability to detect a difference in the two groups. The SF-36v2 is a general health questionnaire. It is possible that a head and neck specific instrument, such as the University of Washington or University of Michigan instruments, may be more sensitive in detecting differences between these two cohorts.

With regard to the comparison between the cancer patients and US normative values, the results were less surprising. Cancer patients scored lower on the SF-36v2 than US norms; however, there were only 4 domains where this decrement was statistically significant. The domains most affected by cancer were physical function, role physical, social function, and role emotional. 

The SF-36v2 instrument has been used in a number of other studies in the head and neck literature. Netscher et al. evaluated the quality of life in patients who had undergone microvascular free flap reconstruction after ablative surgery for advanced oropharyngeal carcinoma [8]. When compared to US norms, these patients had poor perceived quality of life both before and 1 year after surgery with regard to ability to work, vitality, social function, and emotional function. These are the same domains that were significantly lower in our study as well. 

This study is limited in several ways. First, there were a small number of participants in the support group cohort. This problem was inherent to the study design, as participants were recruited from an already existing support group, rather than being invited to participate in the group. There are several other SPOHNC groups in the Los Angeles area, and further studies with other support groups may be possible. Secondly, the two cancer patient cohorts were statistically different with regard to their composition. However, patients were asked to participate without knowledge of their subsite of cancer. Further studies would need subsite and age-matched controls in order to remove this source of bias. It is possible that the preponderance of oropharyngeal cancer patients in the support group cohort diminished the scores in this group and thus negated any SF-36v2 score improvement in this cohort. 

## 5. Conclusions

We failed to demonstrate an improvement in the perceived quality of life of head and neck cancer patients who participate in a head and neck specific support group when compared to those HNC patients who do not participate. However, the camaraderie that exists within a support group may be important to those who do participate and should not be discouraged. Not surprisingly, HNC patients have poorer perceived quality of life when compared to the US population. Further research into ways to improve our patients' posttreatment QOL is important, and further study into support groups or educational programs is warranted. 

## Figures and Tables

**Table 1:** Cohort characteristics.

	Support group cohort	Cancer control cohort	*P* value
Age	60.9	61.4	0.71
Sex (M/F in %)	40/60	50/50	0.55
Location	45% oropharynx	68% oral cavity	0.0036
Time since treatment (in years)	4.4	0.8	0.079

**Table 2:** Comparison with US normative values and cancer cohorts.

Domain	US norms (*Z* scores)	All cancer patients (standard normal distribution = *Z* score)	*P* values (all cancer versus US norms)	Support group patients (mean *Z* scores)	*P* values (support group versus US norms)
Role physical	82.51	60.47	0.0087	56.85	0.006
Physical function	83.29	71.36	0.0003	71.19	0.058
Social function	84.30	67.47	0.0015	67.26	0.013
Role emotional	87.40	68.47	0.0005	63.10	0.003
General health	70.85	64.78	0.101	60.4	0.061

**Table 3:** Comparison of support group cohort versus nonsupport group patients.

Domain	Support group Mean *Z* score	Control group Mean *Z* score	Parametric *P* value
PF	71.19	71.60	0.963
RP	56.85	65.23	0.454
BP	60.62	67.63	0.526
GH	60.40	70.25	0.178
VT	55.36	60.27	0.557
SF	67.26	67.97	0.944
RE	63.1	75.52	0.222
MH	68.81	76.88	0.239
